# Enhancing soil health and strawberry disease resistance: the impact of calcium cyanamide treatment on soil microbiota and physicochemical properties

**DOI:** 10.3389/fmicb.2024.1366814

**Published:** 2024-03-21

**Authors:** Ying-chun Du, Can-sheng Yuan, Yu-qi Song, Ying Yang, Qing-song Zheng, Qiong Hou, Di Wang, Lin Wang

**Affiliations:** ^1^College of Rural Revitalization, Jiangsu Open University, Nanjing, China; ^2^Sanya Research Institute, Nanjing Agricultural University, Sanya, Hainan, China; ^3^College of Resources and Environmental Sciences, Nanjing Agricultural University, Nanjing, China

**Keywords:** continuous cropping obstacle, soil microorganisms, calcium cyanamide, pig manure, network stability

## Abstract

**Introduction:**

Continuous strawberry cropping often causes soil-borne diseases, with 20 calcium cyanamide being an effective soil fumigant, pig manure can often be used as soil organic fertilizer. Its impact on soil microorganisms structure, however, remains unclear.

**Methods:**

This study investigated the effectiveness of calcium cyanamide and pig manure in treating strawberry soil, specifically against strawberry anthracnose. We examined the physical and chemical properties of the soil and the rhizosphere microbiome and performed a network analysis.

**Results:**

Results showed that calcium cyanamide treatment significantly reduces the mortality rate of strawberry in seedling stage by reducing pathogen abundance, while increasing actinomycetes and Alphaproteobacteria during the harvest period. This treatment also enhanced bacterial network connectivity, measured by the average connectivity of each Operational Taxonomic Unit (OTU), surpassing other treatments. Moreover, calcium cyanamide notably raised the levels of organic matter, available potassium, and phosphorus in the soil–key factors for strawberry disease resistance and yield.

**Discussion:**

Overall, applying calcium cyanamide to soil used for continuous strawberry cultivation can effectively decrease anthracnose incidence. It may be by changing soil physical and chemical properties and enhancing bacterial network stability, thereby reducing the copy of anthracnose. This study highlights the dual benefit of calcium cyanamide in both disease control and soil nutrient enhancement, suggesting its potential as a valuable tool in sustainable strawberry farming.

## 1 Introduction

Continuous cropping is a prevalent practice in intensive agriculture, at present, many vegetables and horticultural crops adopt continuous cropping method to save costs, such as tomatoes, peppers. However, this practice can have detrimental effects on soil fertility and physicochemical properties, leading to a decline in crop productivity (Yim et al., 2013; Li X. Y. et al., 2016). This well-known phenomenon is called replant problem, the mechanisms of which are complex, and have been associated with multiple cultural practices, soil conditions and other environmental factors. These conditions are also often associated with accumulated populations of fungal pathogens and nematodes (Seigies and Pritts, 2006). Under continuous rotation, long-term monoculture significantly disturbs soil physicochemical properties, which leads to an imbalance in plant-available nutrients. For instance, prolonged monoculture leads to a decrease in soil pH and diminishes the levels of available nitrogen, phosphorus, and potassium (Tian et al., 2010; Yu et al., 2017). Moreover, replanting accelerates the accumulation of plant autotoxins in continuously cropped species. Allelopathy plays an important role in continuous cropping obstacles (Li H. Q. et al., 2015; Li X. Y. et al., 2015, 2016; Li S. Y. et al., 2016). Toxic substances such as phenolic acids that inhibit the growth of the same plant species have been detected in continuously cropped soils of cucumber, Rehmannia, strawberry and tobacco (Asao et al., 2008; Chen et al., 2011; Li et al., 2012).

Owing to their ability to respond sensitively to environmental changes, soil microorganisms that play significant roles in many processes of soil ecosystems are used as ideal biological indicators of soil health and quality (Brussaard et al., 2007). Studies have shown that long-term continuous cropping can affect soil microbial community structure and diversity, for example, long-term continuous cropping can reduce the size of beneficial soil microbial communities and increase the size of harmful microbial communities (Wu et al., 2007; Caporaso et al., 2012; She et al., 2017). In three soils continuously cropped with tobacco at different for different durations, bacterial diversity and abundance of beneficial bacteria significantly decreased (She et al., 2017). Similarly, continuous cropping of konjac and potato altered the structure and diversity of the soil bacterial community (Liu X. et al., 2014; Wu et al., 2017). Continuous cropping usually results in the enrichment of soil-borne plant pathogens such as Fusarium in rhizosphere soils (Caporaso et al., 2012; Yang et al., 2012).

Strawberry (Fragaria ananassa Duch.) is one of the typical annual plants and terribly threatened by replant problem when new seedlings are established on sites under continuous-cropping condition. Replant problem manifests in stunted growth, declined crop vigor, weak root systems, and drying foliage, all of which lead to low productivity and shortened economic life (Seigies and Pritts, 2006). For continuously cropped strawberry, replant problem has been attributed to biotic and abiotic factors, including accumulated phytotoxic allelochemical substrates (Asao et al., 2008), build-up of specific pathogenic microorganisms (fungi, bacteria and actinomycetes) (Zhen et al., 2005; Nam et al., 2009) and plant-parasitic nematodes, as well as unbalanced availability of plant nutrients and other declines in soil health (soil acidification, soil compaction.).

Calcium cyanamide and pig manure have been reported to be used in continuous cropping barrier soil improvement, but their impact on soil bacterial communities is unknown (Ma et al., 2013; Meng et al., 2021; Chen et al., 2022). As widely acknowledged, the introduction of organic fertilizers dramatically alleviates the soil acidification process in multiple continuous cropping systems, including tobacco, which would reshape the soil fungal community (Dai et al., 2021; Shen et al., 2021). Previous reports indicated that the boost of fungal pathogens in the continuous cropping field was likely due to the depletion of organic C and the increase in N content, which was reversed by organic fertilization (Aguilar et al., 2017). Despite these findings, the effect of calcium cyanamide combined with organic fertilization on the fungal community in continuously cultivated strawberry soil has never been investigated to our best knowledge. Therefore, we investigated the application effects of calcium cyanamide and pig manure in continuously cropped strawberry soil. Through field experiments, we found that the calcium cyanamide treatment significantly reduced the incidence of strawberry and the quantity of anthracnose pathogens (Supplementary Figure S1). We hypothesized that this might be related to changes in the microbiota. To validate our hypothesis, we conducted high-throughput sequencing and determined the physicochemical properties of the soil, analyzing the obtained data.

## 2 Methods

### 2.1 Field experiment description

This study was conducted from July 11, 2022, to March 2023, at the Xiaozhang Strawberry Picking Garden in Hengxi Town, Jiangning District, Nanjing City, Jiangsu Province, China (31.717, 118.762). The experimental site was a continuously cropped strawberry field for over 10 years. A greenhouse was selected for the experiment and divided into 12 plots, each with an area of 40 square meters. Soil treatments began on July 11 and concluded on August 20, lasting for 40 days. The treatments were as follows: CK (untreated), C (calcium cyanamide 60 kg/hm^2^), M (pig manure 500 kg/hm^2^), CM (calcium cyanamide 60 kg/hm^2^, pig manure 500 kg/hm^2^). After uniform application of materials, the soil was plowed evenly, watered, and covered with plastic film to maintain a high-temperature greenhouse environment (The temperature remained at 40–65 degrees throughout the period). The strawberry variety used was “Hongyan,” provided by Nanjing Golden Manor. The distribution of each treatment in the greenhouse is shown in Supplementary Figure S2A. For strawberries, seedlings of uniform size are selected, and 400 seedlings are planted per 40 square meters.

Before planting, the greenhouse had been continuously cultivating strawberries for 10 years. The basic physicochemical properties of the soil were as follows: pH 4.6, electrical conductivity 276 mS/cm, total nitrogen 1.18 g/kg, total phosphorus 1.06 g/kg, total potassium 25.15 g/kg, aviable nitrogen 101.63 mg/kg, available potassium 83.43 mg/kg, available phosphorus 191.06 mg/kg, and organic matter 13.88 g/kg. The nutrient content of pig manure was pH 6.54, electrical conductivity 1932.5 mS/cm, total nitrogen 11.49 g/kg, total phosphorus 1.99 g/kg, total potassium 3.6 g/kg, and total carbon 169.93 g/kg. The calcium cyanamide (provided by Ningxia Darong Chemical Metallurgical Co., Ltd.) had an effective component content exceeding 50%. Strawberry incidence statistics were carried out at the seedling stage (calculated based on the number of deaths and the total number, 400 strawberries of the same size in each plot were planted).

### 2.2 Sample collection, DNA extraction, and chemical property determination

After the completion of plastic film coverage, soil samples were collected from the air-dried soil, referred to as the post-disinfection soil (hereinafter denoted as the C period), after air-drying for 5 days. Strawberries were planted on September 1, and rhizosphere soil samples were collected during the flowering period of strawberries (November 10, denoted as B period) and the fruiting period (December 20, denoted as F). In each treatment, five sampling blocks were selected using a five-point method within the plot (Supplementary Figure S2B), and they were mixed to form one sample. A total of 36 samples were obtained, with three replicates for each of the four treatments across three periods. The soil samples were divided into two parts: one part was immediately frozen at −80°C for DNA extraction, and the other part was air-dried for soil chemical property analysis.

Total genomic DNA was extracted from 0.5 g of soil using the PowerSoil DNA Isolation Kit (MoBio Laboratories Inc., Carlsbad, United States) and dissolved in 50 μL of sterile distilled water following the manufacturer’s instructions. The concentration and quality of the extracted DNA were measured using a NanoDrop 2000 spectrophotometer (Thermo Scientific, United States). The number of anthracnose pathogens in the soil after disinfection was quantified using q-RTPCR. Primers are as follows: LOE-F1: GGCGGG TAGTAGGGTCYCCG, GLOE-R2: ACTCAGAAGAAACGTCGTT AAATCAG.

Basic chemical properties of the soil samples were determined according to the method described by Shen et al. (2013). In brief, soil pH and EC was measured using a glass electrode meter with a soil-to-water ratio of 1:5 (w/v). Soil organic matter (SOM) was determined using the potassium dichromate external heating method. Aavailable potassium (AK), available phosphorus (AP) and available nitrogen (AN) were measured following previous protocols (Cui et al., 2023).

### 2.3 MiSeq sequencing and sequence data processing

The V3-V4 hypervariable regions of the bacterial 16S rRNA gene were amplified from the soil genomic DNA using the primer sets 341F (5′-CCTACGGGNGGCWGCAG-3′) and 806R (5′-GGACTACH VGGGTWTCTAAT3′). The libraries were sequenced on an Illumina MiSeq platform at Majorbio Bio-Pharm Technology Co. Ltd. (Shanghai, China). Raw reads were processed using a custom-developed bioinformatics pipeline whose command-line based script is provided in Hartman et al. (2018). Reads were pre-quality filtered and trimmed at the 3′-end to 280 bp using PRINSEQ (Schmieder, 2011) and then merged with FLASH (Magoc and Salzberg, 2011). Sequences were demultiplexed using Cutadapt (Martin, 2011) and were quality-filtered with PRINSEQ. For operational taxonomic unit (OTU) delineation the 16S rRNA gene sequences were trimmed to the fixed length of 360 bp, sorted by abundance, dereplicated, and clustered to OTUs (≥ 97%, singletons removed) with UPARSE (Edgar, 2013). Chimeric sequences were screened using UCHIME (Edgar et al., 2011) against the GOLD database (Reddy et al., 2015) and removed. Taxonomy assignment was performed using the SILVA database (v119; Quast et al., 2013) with the RDP classifier as implemented in QIIME (Caporaso et al., 2010). Data analysis in R All statistical analyses were conducted in R v3.3.0 (R Core Team, 2015). All raw sequence data have been made available in the NCBI Sequence Read Archive (SRA) database under the access number PRJNA1047888.

### 2.4 α- and β-diversity analysis

Analysis of the α-diversity (the numbers and abundances of taxa within communities) and β-diversity (compositional dissimilarity between communities) metrics were carried out using the vegan package, and the diversities were visualized by using the ggplot2 package in R v4.0.2. Principal coordinate analysis (PCoA) was performed on the distance matrices. Analysis of variance (PERMANOVA, 999 permutations) was performed to evaluate the significant differences among the microbial compositions of the samples. For the separate in-depth analyses of each sample type, we additionally applied the following sequence count threshold to the OTU (Operational taxonomic unit) tables: we selected OTUs with at least two sequences (avoiding singlecount OTUs) that were present in at least six samples (the number of replicates per treatment). We considered OTUs remaining after this thresholding step to represent the soil communities. We normalized the communities using the trimmed mean of Mvalues (TMM) method and expressed the values as relative abundance counts per million (CPM).

### 2.5 Identification of sensitive OTUs and co-occurrence networks

Sensitive OTUs were identified according to the method of Hartman et al. (2018). Briefly, we conducted indicator species analysis with the R package indicspecies (De Cáceres et al., 2010) to calculate the correlation coefficient (r) of each OTU’s positive association with rice cropping patterns. The analysis was conducted with 9,999 permutations and was considered significant at *p* < 0.05. We then tested for differential OTU abundances between one or more of the growth stages of the two cropping communities using likelihood ratio tests with the R package edgeR (Robinson et al., 2010). OTUs whose abundances were identified as differing among growth stages of the two cropping patterns at a false discovery rate (FDR)-corrected value of *p* < 0.05 were considered to be cropping pattern-responsive. Finally, we defined OTUs that were confirmed by both the indicator species analysis and likelihood ratio tests as cropping-sensitive OTUs (sensitive OTUs). Furthermore, we constructed two types of networks: one visualized the significant (*p* < 0.05) OTU associations with different cropping patterns from the indicator species analysis using bipartite networks, and the other utilized the TMM-normalized CPM counts to calculate Spearman rank correlations between OTUs and visualized the positive, significant correlations (r > 0.8 and *p* < 0.01). In the second cooccurrence network, we calculated the network properties, including the total number of network nodes (representing OTUs), the total number of edges (connections between nodes representing positive, significant correlations between OTUs), and the degrees of co-occurrence (number of direct correlations to a node); additionally, we identified the network modules, which are substructures of nodes with higher edge densities within groups than between groups, with the greedy optimization of modularity algorithm implemented in the R package igraph. All networks were visualized with the Fruchterman-Reingold layout with 9,999 permutations and were implemented in igraph (Clauset et al., 2004).

### 2.6 Functional prediction of microbial communities and their correlations with soil nutrients, data analysis

Functional annotations of prokaryotic taxa were carried out using FAPROTAX v.1.1 (Louca et al., 2016). We computed Mantel correlations to evaluate the correlation between co-occurrence network modules using the vegan R software package (Sunagawa et al., 2015). Partial Mantel tests were also performed between the modules and the basic chemical properties of the soils. The data were presented as mean with standard deviation (SD). The data was analyzed using R-studio. When an analysis consisted of only a control and an experimental group, an independent t-Test was performed. When three or more groups were compared, one-way ANOVA was performed followed by a Duncan’s Test. The data were considered significantly different at *p* < 0.05.

## 3 Results

### 3.1 Soil microbiota

**Figure 1:**
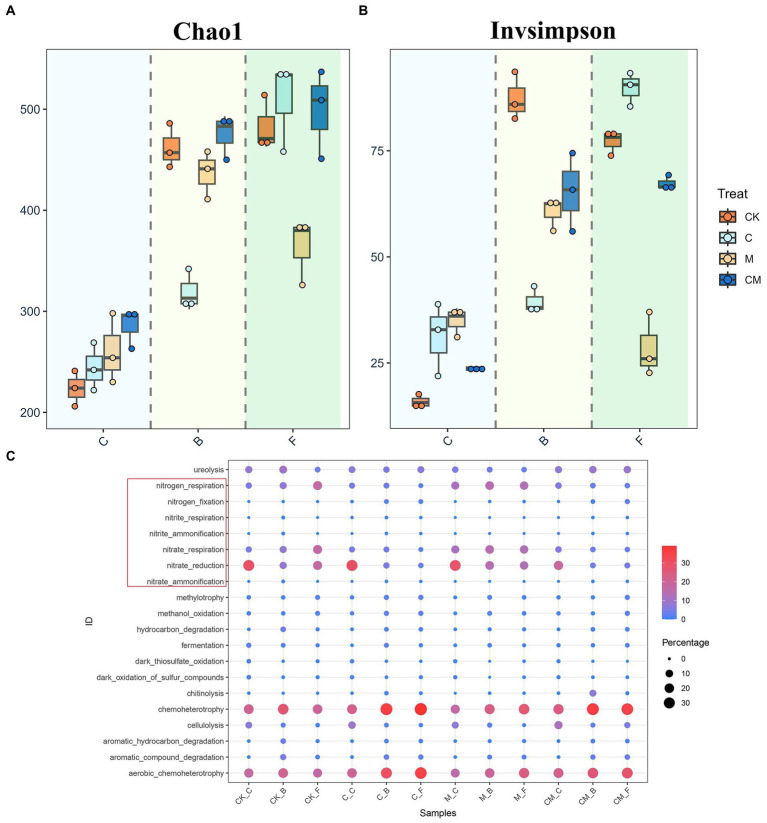
Microbial communities of different treatment. **(A)** Bacterial richness [Chao1, **(A)**] and diversity [**(B)**, inverse Simpson] in soil samples representing different treatment in different growth stages. **(C)** Functional predictions of OTUs in each growth period under different Treatment based on FAPROTAX database retrieval. The red box indicates nitrogen-related functions. Stages: _C After soil disinfection, _B Strawberry blooming period, _F Strawberry fruiting period. Treatment: CK_ Soil without any treatment, C_ Calcium cyanamide treated soil, M_ Soil treated with pig manure, CM_ Soil treated with Calcium cyanamide and pig manure.

In this study, we explored the soil microbial community in strawberry rhizospheres. α-Diversity analysis showed that plant presence led to higher bacterial richness and diversity, peaking in the early growth stage and diminishing over time (Figures 1A,B). Treatment group C had significantly lower richness and diversity in the B-stage but the highest during fruiting. M group’s richness was lowest during fruiting, with no differences in others. CK group showed the highest diversity during flowering, and M group the lowest during fruiting. FAPROTAX database results revealed that microbial richness and diversity changes affected community function diversity (Figure 1C). Nitrogen-fixing functions were significantly higher in the C and CM (calcium cyanamide-treated) groups during B- and F-stages, whereas nitrogen respiration was highest in the CK and M (untreated) groups. The CM group consistently showed higher urease activity and cellulose degradation across all stages. Nitrate reduction and respiration functions were significantly lower in the C and CM groups, indicating reduced nitrate reduction capacity with calcium cyanamide treatment, but these groups excelled in nitrogen fixation and nitrite reduction to ammonia. Conversely, the CK and M groups performed better in nitrogen and nitrate respiration functions without calcium cyanamide.

### 3.2 Impact of different treatments on soil microbial communities

**Figure 2:**
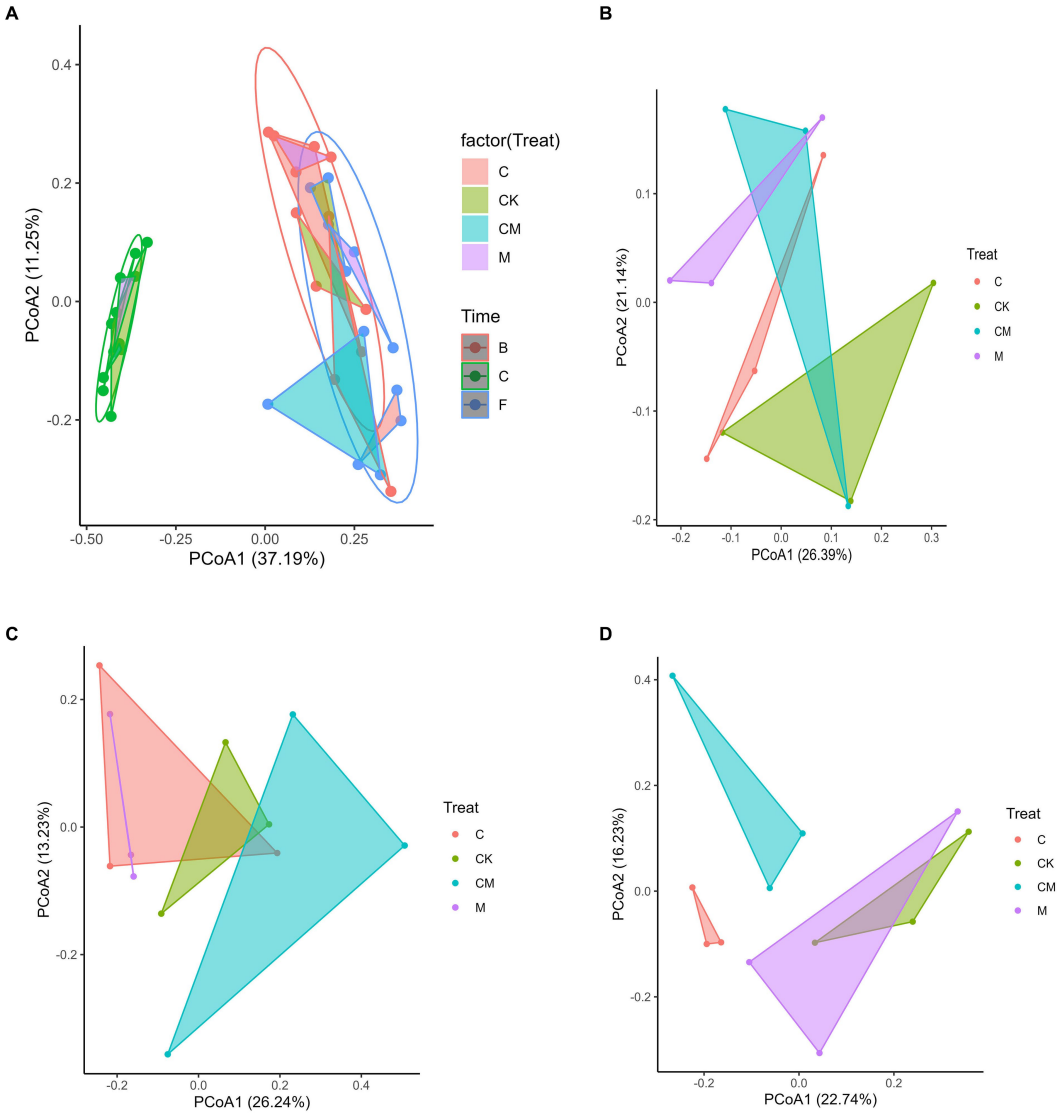
Effects of different treatment on microbial communities. Separate principal coordinate analysis (PCoA) ordinations using Bray-Curtis distance were performed for bacteria. The percentage of variation given on each axis refers to the explained fraction of total variation. Stages: _C After soil disinfection, _B Strawberry blooming period, _F Strawberry fruiting period. Treatment: CK_ Soil without any treatment, C_ Calcium cyanamide treated soil, M_ Soil treated with pig manure, CM_ Soil treated with Calcium cyanamide and pig manure.

To evaluate the effects of various treatments on soil and rhizosphere microbial communities during strawberry cultivation, we performed Principal Coordinate Analysis (PCoA) and Permutational Multivariate Analysis of Variance (PERMANOVA). Our findings revealed significant differences in microbial composition between post-disinfection and strawberry-planted soils, highlighting the impact of strawberry cultivation on rhizosphere microbes (Figure 2A). No notable differences were observed between the flowering and fruiting stages, indicating that strawberry cultivation was the main factor influencing microbial diversity. Distinct microbial separations in post-disinfection soils were noted among the CK, C, and M treatment groups, with the CM group not showing clear differentiation (Figure 2B). Significant microbial variations were observed among the CK, M, and CM groups during flowering (Figure 2C), and between the CK, C, and CM groups at harvest (Figure 2D). This suggests that while treatments affected bacterial communities, strawberry cultivation was the predominant factor altering these communities over time.

### 3.3 Identification of sensitive operational taxonomic units (OTUs)

**Figure 3:**
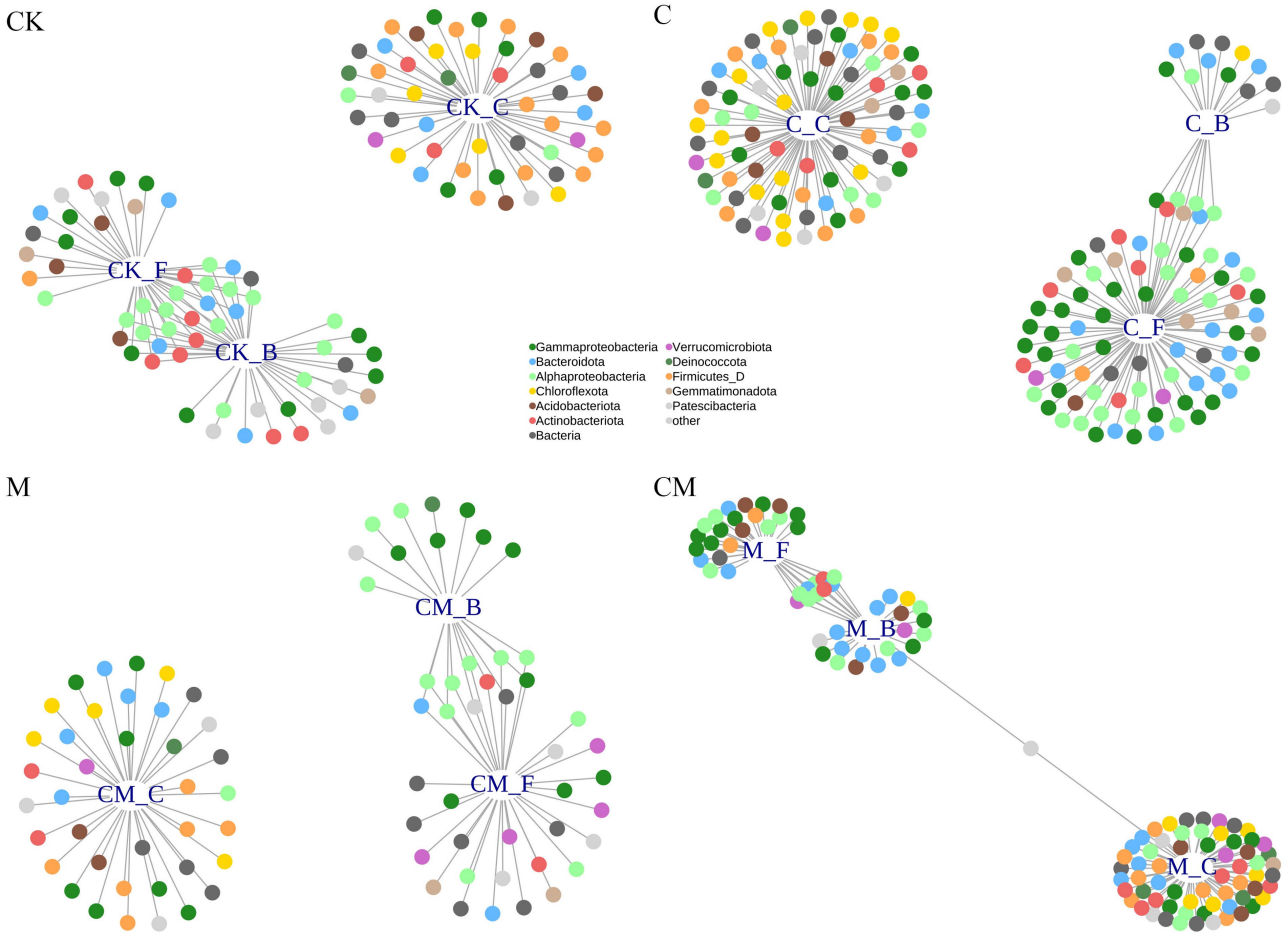
Identifying cultivation-sensitive OTUs. Bipartite networks display sensitive specific OTUs in the different treatment as determined using indicator species analysis. The connecting lines represent individual bacterial OTUs that were positively and significantly associated (*p* < 0.05) with one or more of the growth stages. OTUs are colored according to their phylum assignments. Stages: _C After soil disinfection, _B Strawberry blooming period, _F Strawberry fruiting period. Treatment: CK_ Soil without any treatment, C_ Calcium cyanamide treated soil, M_ Soil treated with pig manure, CM_ Soil treated with Calcium cyanamide and pig manure.

Through indicator species analysis and a bipartite network (Figure 3; Supplementary Table S2), we identified bacterial OTUs that varied in abundance across treatments after strawberry cultivation, revealing significant bacterial clustering during the flowering and fruiting stages. Validation using the likelihood ratio test with EdgeR (Supplementary Figure S4) confirmed cultivation-sensitive OTUs (csOTUs), with 62, 103, 60, and 30 csOTUs identified in the CK, C, M, and CM treatments, respectively, comprising 2.2, 3.7, 2.2, and 1.1% of soil community sequences. This variability underscores the differential impact of treatments on microbial communities at various growth stages.

Despite the substantial overlap in csOTUs across treatments, less than one-third were shared, indicating that while csOTUs responded to cultivation stages, they did not align with specific taxonomic patterns for these stages (Supplementary Figures S5, S6). Each growth stage thus fostered unique bacterial subsets per treatment, though the broader community largely remained consistent across different treatments and stages, emphasizing that specific stages favor unique subsets, yet a core community persists through various treatments and periods.

### 3.4 Impact of different treatments on microbial symbiotic patterns

**Figure 4:**
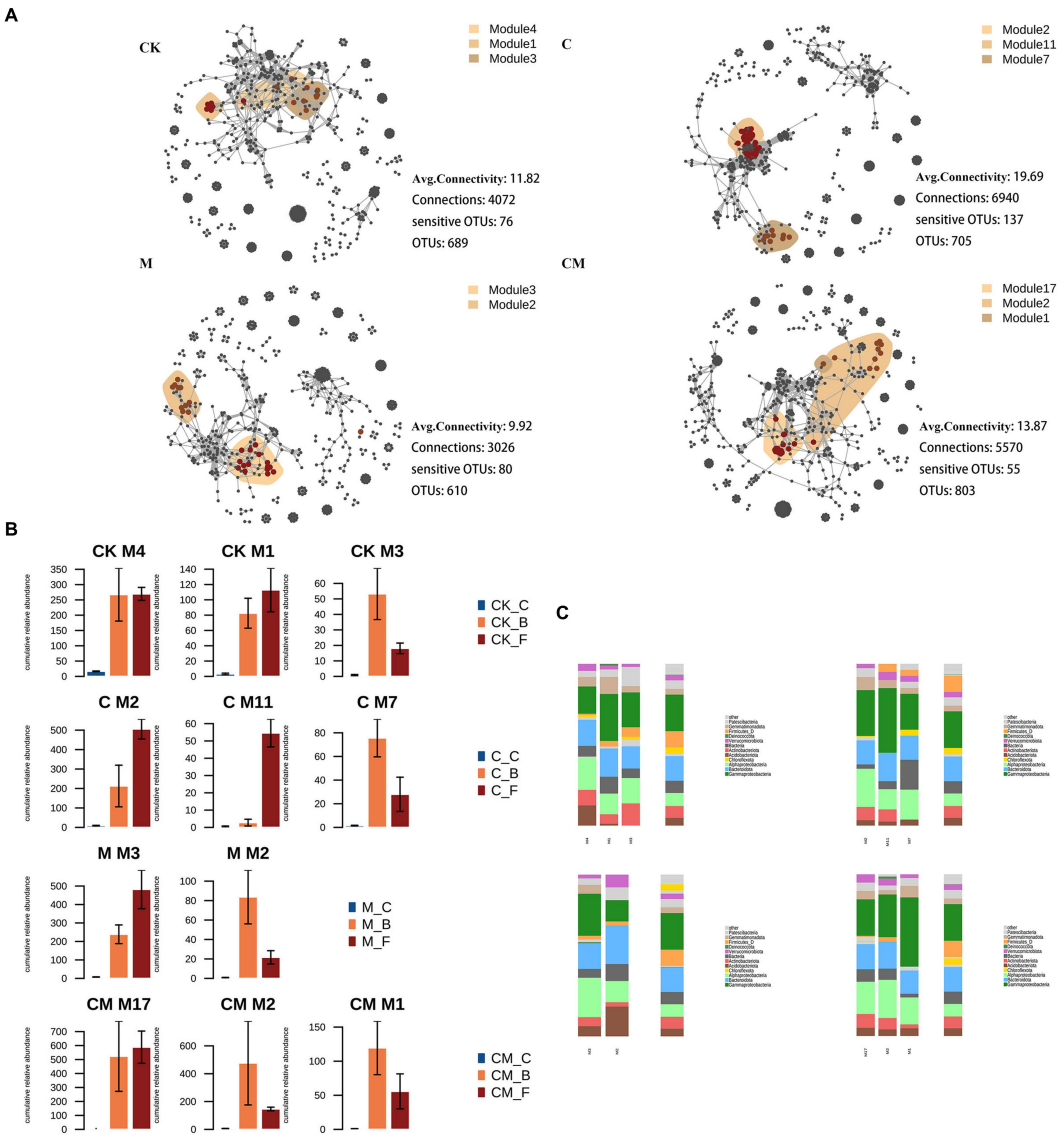
Co-occurrence patterns of cultivation sensitive OTUs. **(A)** Co-occurrence networks visualizing significant correlations (ρ > 0.7, *p* < 0.001; indicated with gray lines) between bacteria OTUs in soil communities. Circles indicate bacteria, and keystone OTUs are represented with asterisks (Supplementary Table S3). OTUs are colored by their association to the different treatment. Shaded areas represent the network modules containing csOTUs of all bacteria of the treatment sensitive modules in soil networks. The cumulative relative abundance in samples of different treatment indicates the overall response of treatment sensitive modules to the different stages. **(B)** Cumulative relative abundances (as counts per million, CPM; y-axis in ×1,000) of the modules in the different treatment networks. **(C)** Qualitative taxonomic composition of cultivation sensitive modules is reported as proportional OTUs numbers per class (bacteria) and compared to the overall taxonomic distribution in the entire dataset (column “all”) stages. OTUs are colored according to their phylum assignments. Stages: _C After soil disinfection, _B Strawberry blooming period, _F Strawberry fruiting period. Treatment: CK_ Soil without any treatment, C_ Calcium cyanamide treated soil, M_ Soil treated with pig manure, CM_ Soil treated with Calcium cyanamide and pig manure.

In our study, we analyzed co-occurrence networks of bacterial Operational Taxonomic Units (OTUs) across various treatments and growth stages, illustrated in Figure 4. The C treatment showed the most significant co-occurring OTUs, suggesting the highest network connectivity compared to M, CK, and CM treatments, with CM having the least. This indicates a stronger microbial network stability in the C treatment. We also integrated cultivation-sensitive OTUs (csOTUs) into these networks, observing distinct clustering patterns according to treatment (Figure 4C).

Analyzing csOTUs’ distribution within these networks highlighted differential microbial association patterns responsive to treatment methods. For example, in the CK treatment, certain modules (M1, M3, M4) contained high csOTU proportions with varied bacterial phyla representation, indicating no clear separation among these modules. Conversely, the C treatment identified modules (M7, M2, M11) with distinct Deinococcota and Actinobacteria proportions, suggesting specific module sensitivity to this treatment. The M and CM treatments showed fewer csOTUs, with M treatment’s modules (M2, M3) showcasing clear separation and a mix of Deinococcus, Bacteroidetes, and Alphaproteobacteria. The CM treatment’s modules (M1, M2, M17) also reflected a similar bacterial composition, indicating treatment-specific sensitivity (Figure 4B). This modular distribution and abundance change across treatments underscore the significant impact of strawberry cultivation on bacterial symbiotic patterns, connectivity, and community dynamics.

### 3.5 Relationship between bacterial abundance and soil nutrients

**Figure 5:**
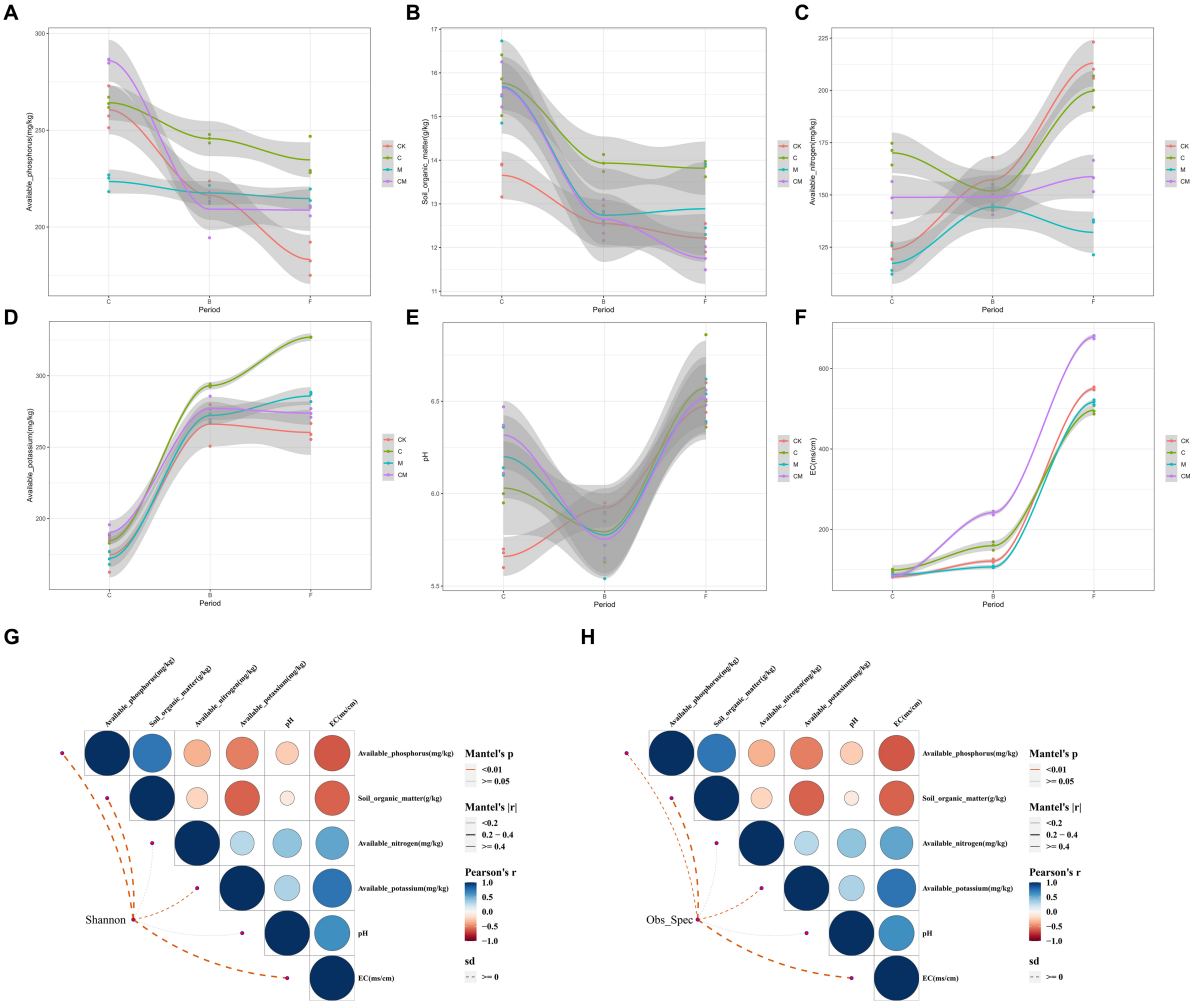
Relationship between co-occurrence network modules and soil nutrients. **(A)** Soil nutrient change trends in different treatment soils during each growth period. **(B)** Pairwise comparisons of soil nutrients and co-occurrence network modules are shown, with color gradients representing Spearman correlation coefficients. Co-occurrence network modules were associated with each soil nutrient through Mantel testing. Edge width corresponds to the Mantel r statistic of the corresponding distance correlation, and edge color indicates statistical significance based on 9,999 permutations. Stages: _C After soil disinfection, _B Strawberry blooming period, _F Strawberry fruiting period. Treatment: CK_ Soil without any treatment, C_ Calcium cyanamide treated soil, M_ Soil treated with pig manure, CM_ Soil treated with Calcium cyanamide and pig manure.

In our study, we examined the relationship between soil nutrients and microbial networks, focusing on key soil nutrients (Figure 5). Post-disinfection, the calcium cyanamide treatments (C and CM) exhibited significantly higher available nitrogen compared to treatments without it (Figure 5C). Throughout the growth cycle, available phosphorus decreased across all groups, with C treatment maintaining higher levels during the flowering and fruiting stages, and CK showing the lowest levels eventually (Figure 5A). Organic matter content was consistently highest in C, followed by M, with CK having the lowest levels (Figure 5B). The C treatment also had higher readily available potassium, crucial for strawberry flowering and fruiting.

Soil pH in the CK group was lower, with no significant differences noted among groups during the flowering and fruiting stages. Electrical conductivity increased over time across all treatments, with CM exhibiting the highest levels, possibly influenced by pig manure (Figure 5D).

Analyzing microbial abundance, diversity, and soil physical and chemical properties revealed no clear correlation (Figures 5G,H), suggesting further investigation is needed. Electrical conductivity showed a strong correlation with readily available phosphorus, and pH correlated with available nitrogen and potassium but not with organic matter. There was no significant relationship between the availability of potassium, nitrogen, and organic matter, highlighting complex interactions between soil nutrients and microbial communities.

## 4 Discussion

Continuous cropping obstacles of strawberry usually create a special soil environment, creating suitable breeding sites for soil-borne diseases and providing abundant hosts, resulting in huge changes in the types and quantities of soil microbial communities (Caporaso et al., 2012; She et al., 2017; Wu et al., 2017). At the same time, the physical and chemical properties of the soil change, the distribution of nutrients is uneven, and the soil becomes compacted (Li H. Q. et al., 2015; Li X. Y. et al., 2016; Li et al., 2018). The research results of this article confirm for the first time that the use of calcium cyanamide can reduce the number of soil pathogenic bacteria and increase the stability of soil microecology on the basis of improving the soil environment, thereby reducing the incidence of strawberry on continuous cropping soil.

### 4.1 Differential responses of microbial communities after different treatments

Based on α- and β-diversity results, including specific sets and functional predictions, we found that strawberry soil after continuous cropping will form different microbial communities when strawberries are planted again after different treatments. First, microbial community richness and diversity decreased after soil disinfection, which is consistent with previous research results (Yang et al., 2023). At the same time, this change continues to weaken as the plants are planted and maintained during the growth period. Until it eventually reaches an equilibrium, this slow process of change will delay the increase in pathogenic species in the soil, leaving more growth time for plants to resist pathogenic bacteria. Of course, changes in richness and diversity cannot fully explain their impact on plant growth. Usually the initial composition of the soil microbial pool is the most important factor in determining the composition of the root microbial community. Past experiments have also shown that tomato-potato intercropping can promote the colonization of Bacillus and protect tomatoes from Verticillium wilt by changing the tomato rhizosphere microbiome (Zhou et al., 2023). The microbiota formed in the early stages can explain the differences in plant growth. We observed microbial differences between different treatments, which may be responsible for plant incidence in later stages. Although we confirmed that the microbial abundance was different in different treatments at different stages, this does not seem to explain the differences in plant growth at later stages, and other analyzes are needed to prove it.

### 4.2 Microorganisms sensitive to different treatments

We identified microorganisms that were sensitive to different treatments and these OTU served as indicator taxa explaining the results of beta diversity. In the C treatment, Gemmatirosa, Bosea, and Paraburkholderia exist as csOTUs. It is reported that Gemmatirosa belongs to a rare bacterial phylum and is a growth-promoting bacterium that can promote the growth of Atractylodes and corn during intercropping (Kong and Liu, 2022; Peng et al., 2022). Bosea has also been reported to be a growth-promoting bacterium isolated from the root nodules of Caragana jubata (Pall.), and there are currently few studies on its function (Pulido-Suarez et al., 2022). These may be weapons to help strawberries fight soil-borne diseases. Calcium cyanamide has been reported to reduce the incidence of soil-borne diseases in vegetables and tomatoes (Chen et al., 2022; Xie et al., 2022). In the M treatment, there is a higher number of cultivation-sensitive OTUs (csOTUs), primarily including members of the phyla Firmicutes, Proteobacteria, Acidobacteria, Bacteroidetes, and Chlorophyta. Many people find that Firmicutes thrive in soils with high biochar content or accept manure. Fertilizer has a higher abundance in the soil (Hartmann et al., 2015; Xu et al., 2017). Chlorophyta is a class of dominant soil microorganisms that obtain nutrients in various ways, such as chemoheterotrophy and photoautotrophy. It is a major participant in soil carbon cycle, nitrogen cycle and sulfur cycle (Gao et al., 2019; Xian et al., 2020). There was no identified csOTU in both the CK and CM groups.

It is reported that adding pig manure to rice fields can increase the abundance of microbial communities that promote soil fertility (Pulido-Suarez et al., 2022). The use of pig manure in continuous cropping cowpea soil can effectively promote the increase in soil enzyme activity (Liu S. et al., 2014). At the same time, pig manure can increase the relative abundance of Sphingomonas, Halobacterium, Nocardia and other bacteria with biological control or plant growth promotion properties in continuous watermelon soil, and enhance the interaction between rhizosphere bacteria. Make the network structure more complex, thereby reducing the occurrence of watermelon wilt disease (Zhang et al., 2022). Our results also indicated a reduced incidence of strawberry anthrax, as well as a reduction in pathogenic, we believe that such results are also related to the structure of the microbial network. However, there is currently no precedent for the combined use of calcium cyanamide and pig manure to improve continuous cropping soil. Although the most effective treatment for reduction was calcium cyanamide, strawberry incidence was also significantly reduced in the pig manure and pig manure combined with calcium cyanamide treatments. This shows that calcium cyanamide treatment has the strongest ability to kill pathogenic bacteria.

### 4.3 Effects of different treatments on the construction of microbial symbiosis network

Our study found significant variations in microbial network complexity among four treatments, with the C treatment displaying the highest connectivity and the CM group the most nodes, suggesting greater microbial richness and diversity. Such complexity often correlates with ecological resilience and stability (Landi et al., 2018). OTUs across different network modules at various growth stages indicates distinct microbial community structures that cluster tightly in response to specific treatments.

Keystone taxa are thought to frequently interact with many other taxa, thereby playing important roles in the overall community (Berry and Widder, 2014; Ma et al., 2016). The study found that although different treatments had effects on beta diversity and network pattern, their effects were mainly limited to non-key taxa, and we found that 5 keystone otu were sensitive to C treatment (Supplementary Table S3). No taxa were sensitive to CK and CM. The M treatment group has 19 keystone OTU. It is important to emphasize that co-occurrence networks visualized relationships between taxa include true ecological interactions (e.g., mutualism), but are also non-stochastic processes (e.g., niche overlap) and, therefore, do not necessarily reflect relationships between taxa (Faust and Raes, 2012; Weiss et al., 2016). Future experiments will assess whether the identified keystone or cultivation-sensitive species directly affect other members of the microbiome or indirectly affect host performance and fitness, thereby affecting other community members. However, co-occurrence networks are a useful tool for exploring abundance patterns in complex microbial communities and may be useful in designing future experiments. For example, combined with reference populations of microbial isolates, plant growth experiments can be performed and the presence/absence or relative abundance of key taxa identified through network analysis can be manipulated and the effects on plant growth and development scored (Schlaeppi and Bulgarelli, 2015). This will also help us understand our experimental results and data more deeply.

### 4.4 The relationship between symbiotic network modules and soil nutrients

The addition of calcium cyanamide will usually lead to an increase in nitrogen content in the soil, and calcium cyanamide will increase available nitrogen (Ma et al., 2013), increase organic matter, available phosphorus and available potassium content. This is consistent with our experimental results. The addition of pig manure usually promotes an increase in organic matter content in the soil (Hou et al., 2022; Zhang et al., 2022), our results also confirm that the increase in organic matter (Lin et al., 2019). Soil nutrient content is usually closely related to microbial communities, and in this study, we found that species richness and diversity may have a certain correlation with soil organic matter, available phosphorus and electrical conductivity, but not with available nitrogen and pH. Of course, the prediction method developed based only on the FAPROTAX database cannot accurately describe the relationship between microorganisms involved in nutrient transformation activities and microorganisms with nutrient transformation functions, but to a certain extent, this result may indicate that in the C co-occurrence module, interactions between species are stronger than in other co-occurring modules, even if their number is not the largest. In addition, this conclusion also expands people’s understanding of the “functional core microbiome.” It is important to emphasize that co-occurrence networks visualize correlations between taxa, including true ecological interactions (e.g., mutualism), but also visualize non-stochastic processes (e.g., niche overlap) and therefore do not necessarily reflect taxa direct interaction between (Faust and Raes, 2012; Weiss et al., 2016).

Future experiments will assess whether the identified keystone or cultivation-sensitive species directly impact other members of the microbiome or indirectly impact host performance and fitness. Microbial communities can then be validated and modulated through isolation, screening, and callback. We will try to combine the special agricultural management model of the C model to establish a soil microbial environment that is conducive to the ecological environment.

## 5 Conclusion

Soil pre-treatment not only alters the physicochemical properties of the soil but also reshapes the soil microbial community. In comparison to traditional soil sterilization methods, adding calcium cyanamide promoted an increase in soil organic matter, readily available potassium, and readily available phosphorus content. This addition enhances the stability of the soil bacterial community throughout the entire strawberry cultivation process, consequently reducing the incidence of strawberry diseases. The rationale behind this might be attributed to the initial disinfection effect of calcium cyanamide on harmful pathogens, leading to a subsequent microbial community recovery that favors beneficial microbes. Compared to treatments involving pig manure and a combination of pig manure and calcium cyanamide, the standalone use of calcium cyanamide exhibits the most effective reduction in the incidence of strawberry diseases. This is likely because the impact of calcium cyanamide on the soil is more pronounced. This study primarily focuses on the network stability across the entire growth cycle of strawberries under different treatments and its correlation with the incidence of strawberry diseases. In future research, we will continue to investigate the variations in microbial communities under different treatments and their relationship with strawberry yield and quality.

## Data availability statement

The data presented in the study are deposited in the National Center for Biotechnology Information(NCBI) BioProject database repository, accession number PRJNA1047888.

## Author contributions

Y-cD: Conceptualization, Methodology, Software, Validation, Formal analysis, Investigation, Resources, Data curation, Writing – original draft. C-sY: Validation, Visualization, Supervision, Project administration, Funding acquisition, Writing – review and editing. Y-qS: Validation, Resources, Writing – review and editing. YY: Software, Data curation, Writing – review & editing. Q-sZ: Validation, Investigation, Writing – review and editing. QH: Formal analysis, Supervision, Writing – review and editing. DW: Methodology, Writing – original draft. LW: Conceptualization, Writing – review and editing.
