# Supplementary material for: Enhancing soil health and strawberry disease resistance: the impact of calcium cyanamide treatment on soil microbiota and physicochemical properties
Source: Front Microbiol. 2024 Mar 21;15:1366814. doi: 10.3389/fmicb.2024.1366814 (PMC10991749; doi:10.3389/fmicb.2024.1366814)
Supplement: Supplementary file 1 [file Presentation_1.zip › Figure legend0228-for Supplem.docx]

FigureS1：Incidence of strawberry at seedling stage and quantification of anthracnose pathogens. （A）Incidence of strawberry at seedling stage（B）Quantification of anthrax pathogens

FigS2: Schematic diagram of experimental. (A). Field layout of soil treatment patterns. (B). Field sampling method.

Figure S3: Unconstrained PCoA ordinations of bacteria. Sample type presented the major driver of community variation. Percentage of variation given on each axis refers to the explained fraction of total variation in the community. Upper and lower panels are colored by sample type. Symbols refer to the different stages.

Stages: _C After soil disinfection, _B Strawberry blooming period, _F Strawberry fruiting period.

Treatment: CK_ Soil without any treatment, C_ Calcium cyanamide treated soil, M_ Soil treated with pig manure, CM_ Soil treated with Calcium cyanamide and pig manure

TableS1

| **Df** | **SumOfSqs** | **R2** | **F** | **Pr(>F)** | **Column1** |
| --- | --- | --- | --- | --- | --- |
| Treat | 3 | 0.729035608 | 0.081584188 | 1.512504972315 | 0.0728 |
| **Period** | **2** | **3.206820218** | **0.35886563** | **9.97961856725652** | **0.0001** |
| Treat:Period | 6 | 1.144092231 | 0.128031929 | 1.18680430008929 | 0.2029 |
| Residual | 24 | 3.85604343 | 0.431518252 | NA | NA |
| Total | 35 | 8.935991487 | 1 | NA | NA |

Results of PERMANOVA testing the effects of Block, Sample type and Cultivation System on bacterial communities. Significant effects are indicated in bold (*p<0.05, **p<0.01, ***p<0.001).

Figure S4: Defining treatment sensitive bacteria in soil samples. Venn diagrams show the number of OTUs res ponding to treatment practices identified with indicator species analysis (purple) and by edgeR (cyan). OTUs identified by both methods were defined as cultivation sensitive OTUs (csOTUs).

Figure S5: Mean relative abundances (counts per millior1, CPM; log2 scale) of cultivation sensitive OTUs (as defined in Fig. S4, summarized at phylum level) across different treatment systems for soil bacteria.

Figure S6: Mean relative abundances (counts per millioln, CPM; log2 scale) of cultivation sensitive OTUs identified by indicator species analysis and edgeR(see Fig.S4).OTUs are labeled with their family level taxonomy assignment, with the phylum level taxonomy assignment indicated by the colored bars.

TableS2. An XLSX table reporting the indicator species and edgeR results and the assignments to cultivation sensitive OTUs and network modules.

TableS3. Characteristics of keystone OTUs.
