# Supplementary material for: Enhancing soil health and strawberry disease resistance: the impact of calcium cyanamide treatment on soil microbiota and physicochemical properties
Source: Front Microbiol. 2024 Mar 21;15:1366814. doi: 10.3389/fmicb.2024.1366814 (PMC10991749; doi:10.3389/fmicb.2024.1366814)
Supplement: Supplementary file 1 [file Presentation_1.zip › TableS1.docx]

| **Df** | **SumOfSqs** | **R2** | **F** | **Pr(>F)** | **Column1** |
| --- | --- | --- | --- | --- | --- |
| Treat | 3 | 0.729035608 | 0.081584188 | 1.512504972315 | 0.0728 |
| **Period** | **2** | **3.206820218** | **0.35886563** | **9.97961856725652** | **0.0001** |
| Treat:Period | 6 | 1.144092231 | 0.128031929 | 1.18680430008929 | 0.2029 |
| Residual | 24 | 3.85604343 | 0.431518252 | NA | NA |
| Total | 35 | 8.935991487 | 1 | NA | NA |

TableS1

Results of PERMANOVA testing the effects of Block, Sample type and Cropping System on bacterial communities. Significant effects are indicated in bold (*p<0.05, **p<0.01, ***p<0.001).
