# Supplementary figures and images for: Enhancing soil health and strawberry disease resistance: the impact of calcium cyanamide treatment on soil microbiota and physicochemical properties
Source: Front Microbiol. 2024 Mar 21;15:1366814. doi: 10.3389/fmicb.2024.1366814 (PMC10991749; doi:10.3389/fmicb.2024.1366814)

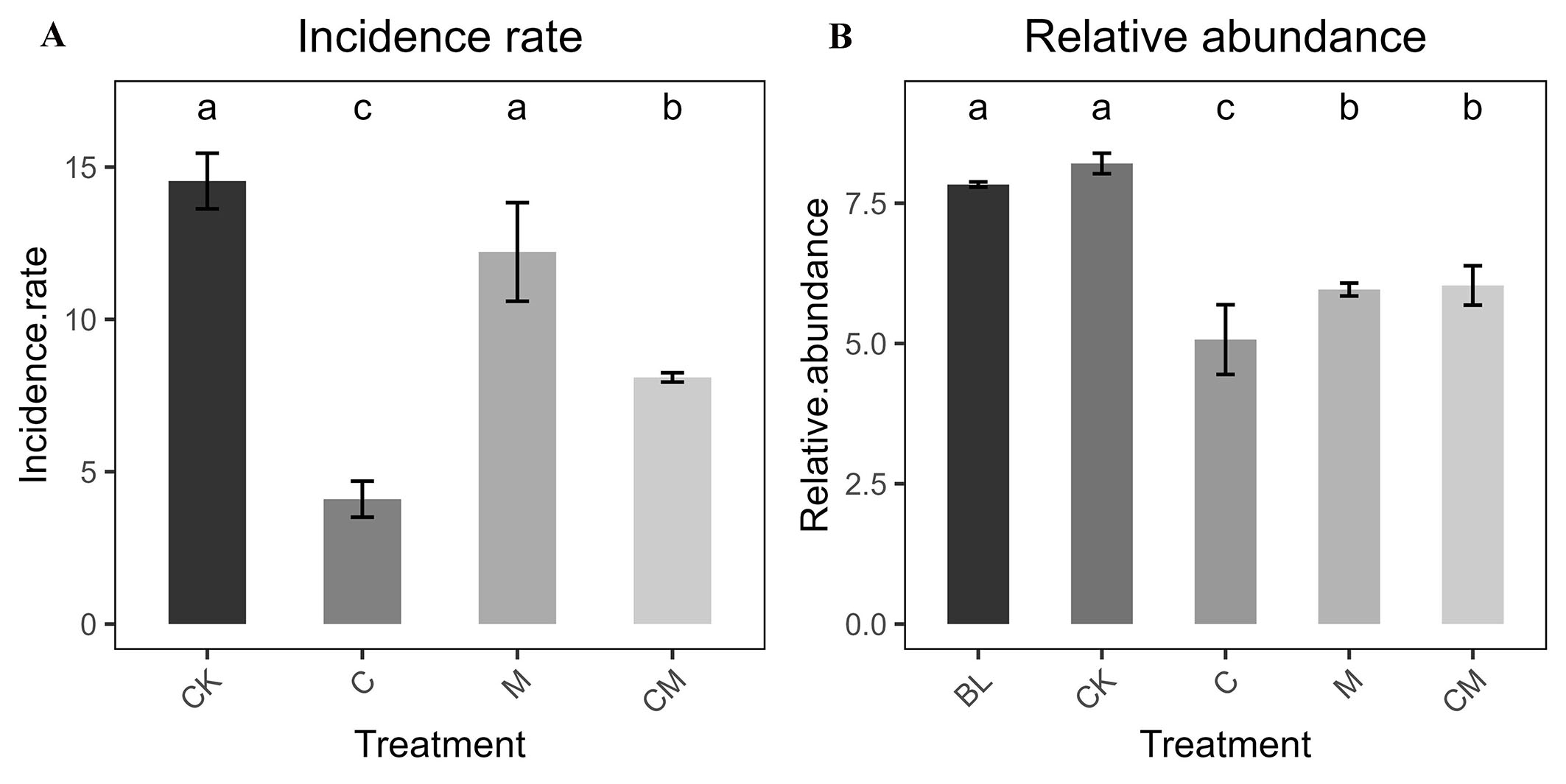

Supplement: Supplementary file 1 [file Presentation_1.zip › FigureS1.jpg]

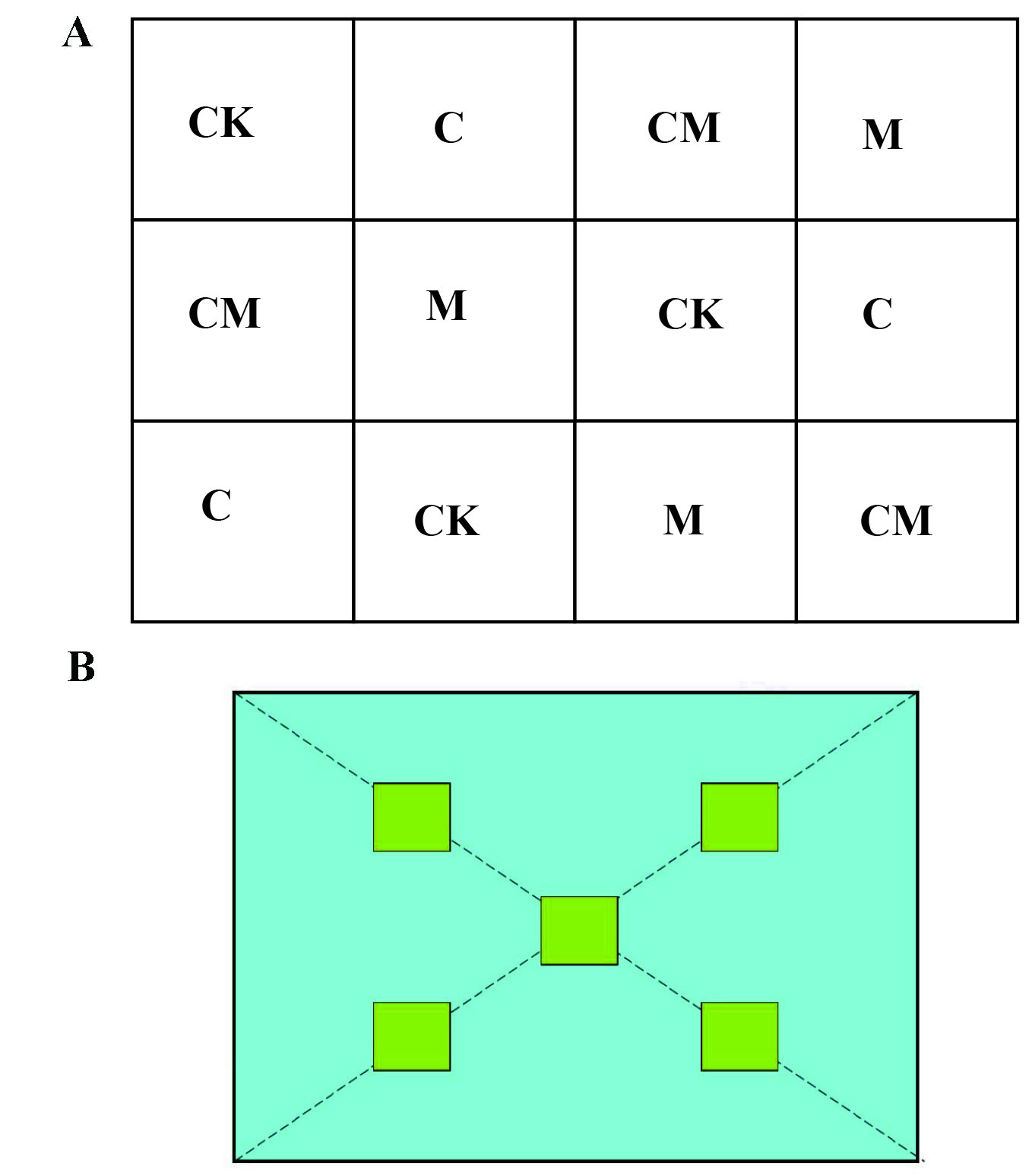

Supplement: Supplementary file 1 [file Presentation_1.zip › FigureS2.jpg]

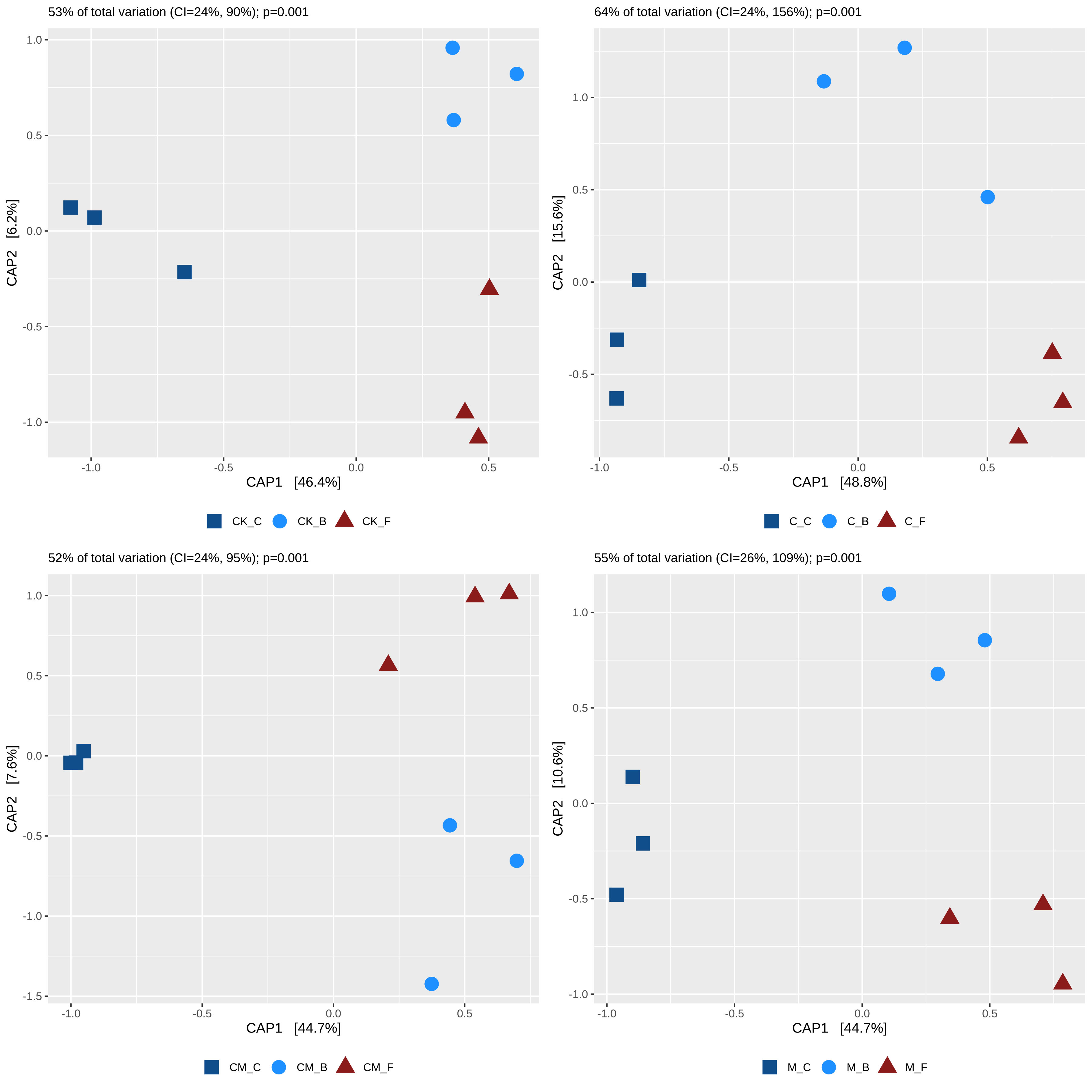

Supplement: Supplementary file 1 [file Presentation_1.zip › FigureS3.jpg]

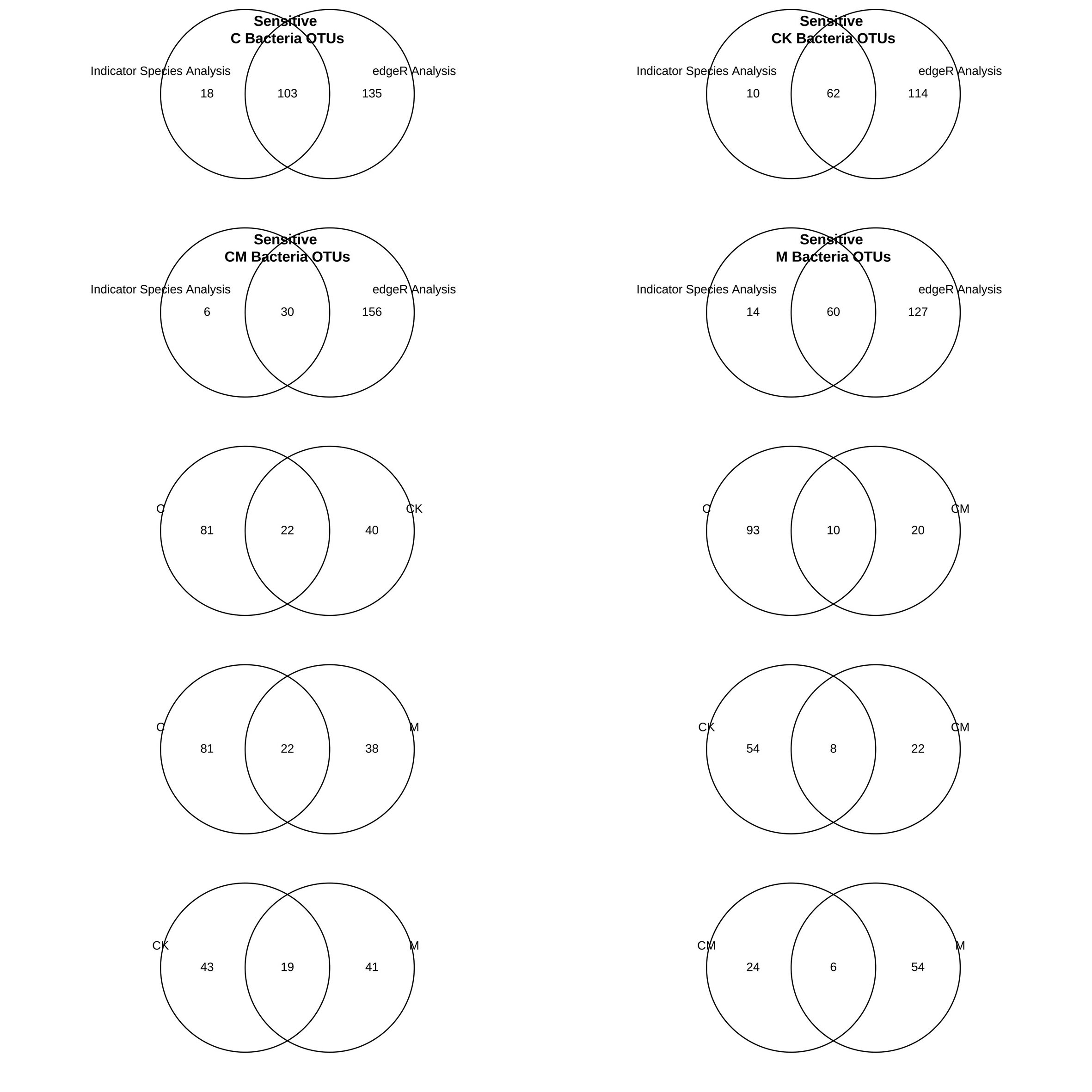

Supplement: Supplementary file 1 [file Presentation_1.zip › FigureS4.jpg]

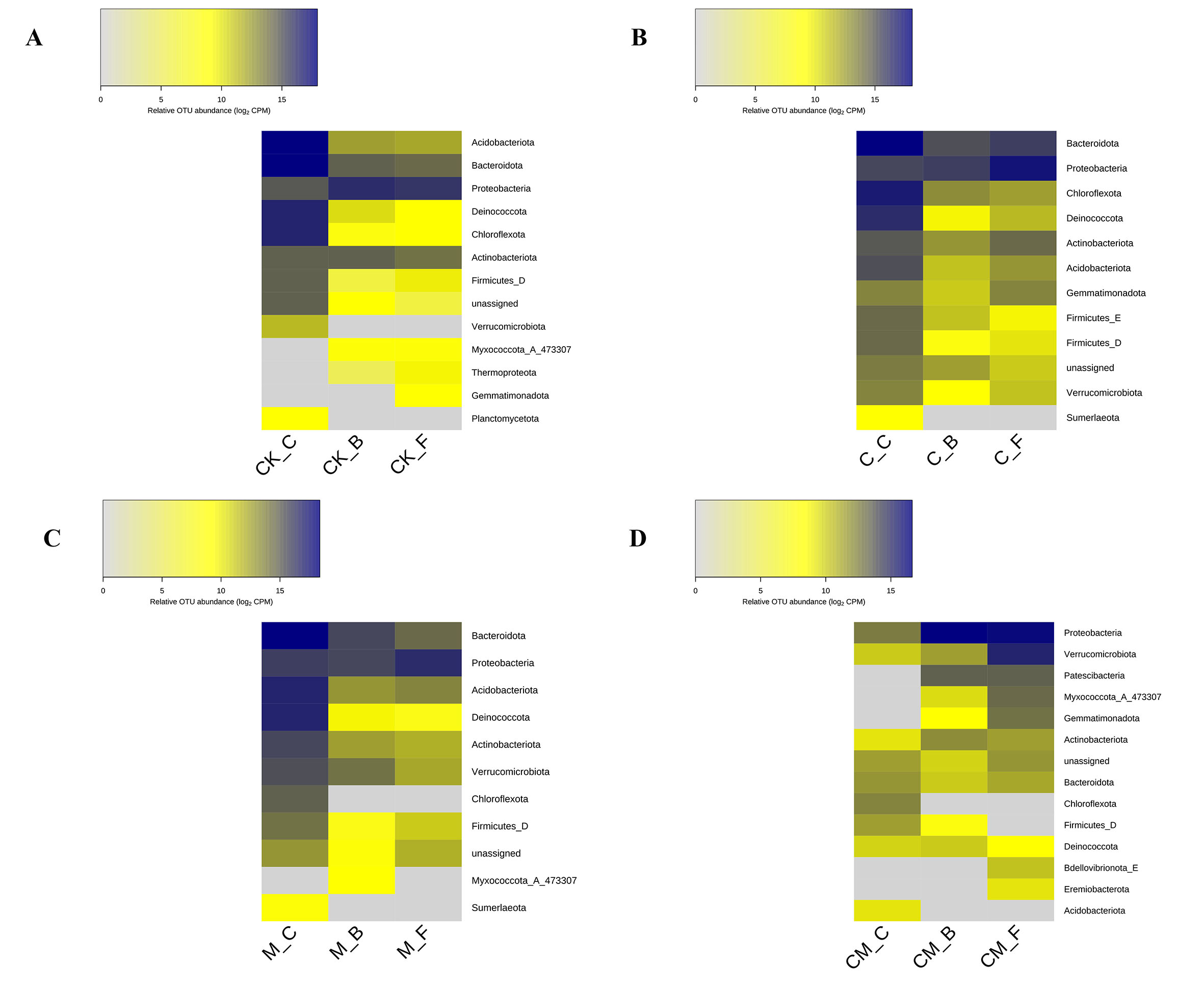

Supplement: Supplementary file 1 [file Presentation_1.zip › FigureS5.jpg]

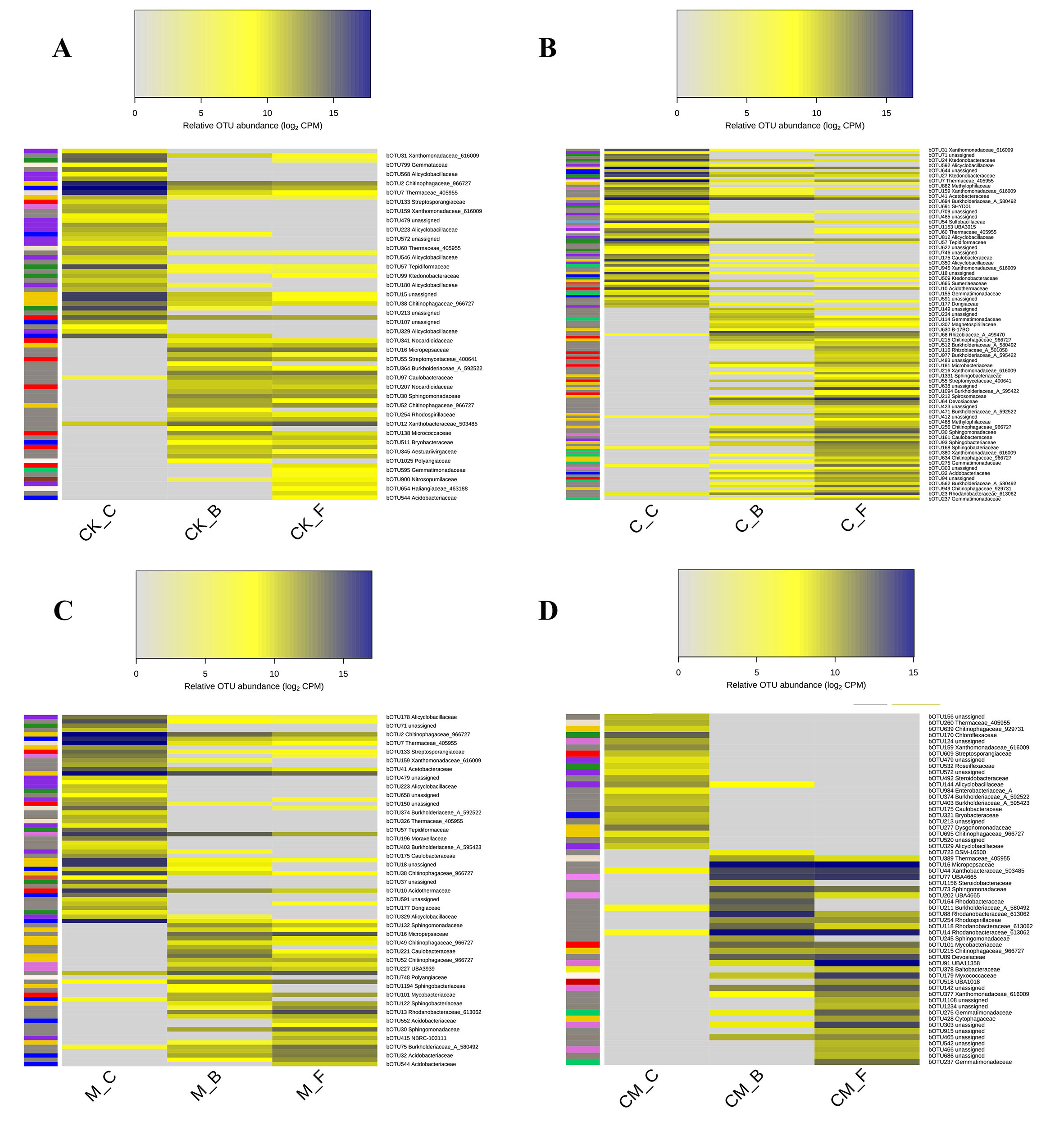

Supplement: Supplementary file 1 [file Presentation_1.zip › FigureS6.jpg]
